# Remedy for Intussusception of the Bowels

**Published:** 1852-11

**Authors:** A. S. Baldwin

**Affiliations:** Jacksonville, Florida; Ky.


From the American Journal of the Medical Sciences.

*Remedy for Intussusception of the Bowels, by* A. S. BALDWIN, of Jacksonville, Florida. (Extract from a Letter to the Editor.)

HAVING recently seen, in the medical periodicals, several reports of *post-mortem* examinations, in cases of intussusception of the bowels, I am induced to send you an account of a rather simple, but what appeared to me a very effectual remedy for this complaint, in a case which came under my care about four years ago, when I had despaired of affording relief by the ordinary remedies. If it shall seem to you to have sufficient merit, it may, perhaps, by giving it publicity in your Journal, induce other members of the profession to give it a trial. During the last fifteen years, several cases of this complaint have come under my observation, most of which terminated fatally. The various remedies recommended were applied; among them the long elastic tube, for the purpose of throwing fluids high up into the bowels, with the hope of distending them so that the vaginated folds of the intestine might be drawn out. In some instances, at least, its use appeared to be productive of mischief, from passing through the constricted part of the intestines, so that the fluid injected was lodged in the pouch or sack existing above the point of obstruction; to ineffectually increase the distension already existing there; for, when the tube was withdrawn, no fluid was returned, and the distension was increased, without having the effect of removing the difficulty.—Injections administered by the common syringe were returned immediately, even while giving them, and had no effect to distend the lower bowel so as to aid in overcoming the obstruction, and the conclusion arrived at was, that adhesion existed between the folds of the invaginated parts of the bowel, and the obstruction was irremediable.

Circumstances which have occurred to me, dispose me to think that these adhesions do not take place so early, or so frequently, as many are disposed to believe. About three years ago, a case of this kind occurred in my practice, and for several days, all the appliances which had been recommended, had been used to overcome the obstruction, but without avail. There was a circumscribed spot, to the left of the umbilicus, and a little below it, which was painful; there was considerable distension of the abdomen, with a sensation of soreness across, but which did not amount to pain,

(being what is called in this country "*misery*.") Twice in this case, the long tube was used, and injections thrown high up into the bowels, which, however, did not return upon the withdrawal of the tube, but added to the swelling previously existing above the point of obstruction. I was apprehensive that adhesion had by this time taken place, and as I despaired of relief by the ordinary methods, and the patient had arranged his temporal affairs, and given himself up to die, I determined to distend the lower bowels, to their utmost capacity, by the injection of warm water.

An ivory tube, having a shield around it, was introduced and passed up until the shield was pressed against the sphincter ani, a cloth was wrapped around this, and pressed up firmly; the tube was now connected by an elastic tube, with the pump, which was placed in a wash-basin of warm water, which was slowly injected into the bowels, pressure being kept up to prevent its return.—Another basin of water was brought, half of which was thrown up. The abdomen was, of course, much distended by this quantity of fluid, and considerable rumbling and commotion of the bowels were produced, the pain at the point of obstruction was, for a moment, acute, causing the patient to cry out. The pressure and the tube were removed, and we found he had the power to retain the injection until he could be helped to the chair, when about five quarts of the injection were passed; becoming faint, he was laid upon the bed, and brandy and water administered; he soon rallied, and passed as much more, colored by fæcal matter; soon after, a copious and regular, but very offensive stool was had, in which the oil, taken several days before, could be distinguished. After this, he had no further difficulty, except debility, and a sensation of soreness at the point of obstruction, which lasted for a few days, when he returned to his work, that of a carriage-maker, and up to the present time has had no return of the complaint. Since that time, I have not had so severe a case of this complaint, but in every case which shows a disposition to be obstinate, I resort to this mode of injection, with uniform and immediate success.—Perhaps some of these would have been as obstinate as the one above detailed, if the former mode of treatment had been pursued; but I am fully impressed with the belief that, had this remedy been used in those cases which had proved fatal, some of them, at least, might have been saved. The case in which I first tried it, was an unpromising one, on account of the long time which had elapsed since the attack, before the remedy was used; sufficient for adhesive inflammation to have agglutinated together the folds of the intestine involved in the intussusception.

Jacksonville, Ky., June 3, 1852.

